# To Our Patrons

**Published:** 1867-07

**Authors:** 


### TO OUR PATRONS.

Our patrons will bear with us, when we assure them, that such is the stringency of the times, that we are obliged to ask them for remittances. We do not see how we can meet current expenses unless our friends will come to our rescue.

Our Kentucky and Tennessee subscribers have generally paid in advance, which have relieved us much, and for which we thank them. Knowing the poverty of our Southern friends, who like ourselves, emerged from the war, homeless, and destitute; and knowing their willingness to pay when able to do so, have not often asked them for any thing, and would not now, but for the fact, that necessity drives us to it.

Amid obstacles that seemed insurmountable, we have, for two years, sent the Atlanta Journal of Medicine and Surgery to our friends. We have labored night and day to do so, and will continue do it, come what may.

But we assure them that we have two thousand dollars and more on our books, that has been earned under difficulties that would have appalled the stoutest heart—a sum sufficient to publish the JOURNAL for one year. The half of it would make us easy, and our patrons would not feel it. Will they pay half, and when the crops are sold pay the balance? Will our friends remember us?

